# Expression of CGRP in the Trigeminal Ganglion and Its Effect on the Polarization of Macrophages in Rats with Temporomandibular Arthritis

**DOI:** 10.1007/s10571-024-01456-7

**Published:** 2024-02-16

**Authors:** Junli Tao, Xiaohui Wang, Jie Xu

**Affiliations:** 1 College of Stomatology, Chongqing Medical University, Chongqing, China; 2 Chongqing Key Laboratory for Oral Diseases and Biomedical Sciences, Chongqing, China; 3 Chongqing Municipal Key Laboratory of Oral Biomedical Engineering of Higher Education, Chongqing, China

**Keywords:** Calcitonin gene-related peptide, Macrophages, Neuroinflammation, Temporomandibular joint osteoarthritis, Trigeminal ganglion

## Abstract

Calcitonin gene-related peptide (CGRP) is synthesized and secreted by trigeminal ganglion neurons, and is a key neuropeptide involved in pain and immune regulation. This study investigates the expression of CGRP in the trigeminal ganglion (TG) and its regulatory role in the polarization of macrophages in rats with temporomandibular arthritis. A rat model of temporomandibular arthritis was established using CFA. Pain behavior was then observed. Temporomandibular joint (TMJ) and the TG were collected, and immunohistochemistry, immunofluorescence (IF) staining, and RT-qPCR were used to examine the expression of CGRP and macrophage-related factors. To investigate the impact of CGRP on macrophage polarization, both CGRP and its antagonist, CGRP 8-37, were separately administered directly within the TG. Statistical analysis revealed that within 24 h of inducing temporomandibular arthritis using CFA, there was a significant surge in CD86 positive macrophages within the ganglion. These macrophages peaked on the 7th day before beginning their decline. In this context, it’s noteworthy that administering CGRP to the trigeminal ganglion can prompt these macrophages to adopt the M2 phenotype. Intriguingly, this study demonstrates that injecting the CGRP receptor antagonist (CGRP 8-37) to the ganglion counteracts this shift towards the M2 phenotype. Supporting these in vivo observations, we found that in vitro, CGRP indeed fosters the M2-type polarization of macrophages. CGRP can facilitate the conversion of macrophages into the M2 phenotype. The phenotypic alterations of macrophages within the TG could be instrumental in initiating and further driving the progression of TMJ disorders.

## Introduction

Approximately 10–15% of adults suffer from temporomandibular arthritis and masticatory muscle dysfunction and pain (Gonçalves et al. 2011). Chronic pain, the primary complaint of temporomandibular disorders (TMD) patients, not only diminishes their quality of life but also imposes significant health issues and a considerable societal burden. Despite its prevalence, the precise cause of TMD pain remains elusive. Recently, neuroimmune factors, which are potential targets for the prevention and treatment of inflammation, have garnered attention in temporomandibular arthritis research (Maruyama et al. 2017).

The TMJ receives its sensory innervation primarily from the auriculotemporal and masseter nerves. Both of these nerves are branches of the mandibular division of the trigeminal nerve (Kucukguven et al. 2022). Structurally, the TMJ is composed of the mandibular fossa, mandibular condyle, and the articular disc, all of which are enveloped by the articular capsule. This capsule comprises an outer fibrous layer and an inner synovial membrane. Within the synovial membrane, two distinct cell types can be identified: fibroblast-like stromal cells and lining cells (Nozawa-Inoue et al. 2003). Moreover, immune cells, such as macrophages, neutrophils, and mast cells, are also present in this membrane (Hayashi et al. 2002; Nozawa-Inoue et al. 2003). It's noteworthy that primary headaches, including migraines and TMD, often involve the trigeminal nervous system in their pathogenesis. Calcitonin gene-related peptide (CGRP), a neuropeptide, has been implicated in the etiology of migraines. Furthermore, its role in the pathogenesis of TMD and the onset of cranial hypersensitivity makes it a promising target for TMD treatments (Cady et al. 2011; Romero-Reyes et al. 2015; Shu et al. 2020).

CGRP, synthesized and secreted by trigeminal ganglion neurons, is a pivotal neuropeptide in pain and immune regulation. Its release from peripheral or central terminals and neuronal cell bodies serves to activate other neurons and satellite glial cells through autocrine or paracrine mechanisms. Notably, during the chronic pain phase of OA, an upsurge in trigeminal ganglion’s CGRP is observed (Duffadar et al. 2009; Miller et al. 2017). This neuropeptide, predominantly generated by neurons, is released from neural endings to exercise its modulatory effects. Upon release, it binds to CGRP receptors, thereby modulating the immune functionalities of macrophages (Ma et al. 2010; Mikami et al. 2013). Further investigations highlight a potential significant association between CGRP-induced macrophage modulation and the pro-inflammatory cytokine TNF-α (Li et al. 2010). Such research underscores the extensive immunoregulatory role of both neurogenic and macrophage-derived CGRP, laying a solid foundation for examining their impacts on the immune response in temporomandibular arthritis.

Recent studies have demonstrated that immune cells, which infiltrate injured nerves, play multifaceted roles in pathological neurons (Stratton and Shah 2016). Macrophages, for instance, are pivotal in regulating inflammation resolution. These immune cells promote tissue homeostasis by transitioning their phenotype from the pro-inflammatory M1 state to the anti-inflammatory M2 state (Alvarez et al. 2016). Despite being a form of neurogenic inflammation, the impact of macrophage phenotypic shifts in the TG on peripheral nerve repair and hyperalgesia during the progression of temporomandibular joint arthritis remains unexplored. To address this gap, we established a rat model of temporomandibular arthritis. This study delves into the polarization of macrophages in the trigeminal nervous system both morphologically and molecularly during arthritis progression. Additionally, we probed the role of macrophage infiltration in the rat’s trigeminal ganglion during temporomandibular arthritis and examined the interplay between CGRP and macrophage polarization. Our findings lay the groundwork for further research into the therapeutic potential of macrophage-targeted treatments for temporomandibular arthritis and offer new insights for neuropathic pain management.

## Materials and Methods

### Animals and Anesthesia

In our study, we acquired 48 seven-week-old male Sprague–Dawley (SD) rats, each weighing between 220 and 270 g, from the Experimental Animal Centre of Chong Qing Medical University. These rats were maintained in a specific pathogen-free (SPF) laboratory environment. Our experimental procedures were thoroughly reviewed and approved by the Ethics Committee for Animal Research at the School and Hospital of Stomatology, Chong Qing Medical University, China, with the given approval number: Ethics Review 2022 (119).

### Establishment of a CFA-Induced TMJ OA Model

After a one-week isolation period, we randomly divided the rats into 8 groups, with each group consisting of 6 SD rats. The first group received saline injections in the temporomandibular joint (TMJ). Groups two to five had CFA injected into the TMJ, with subsequent observations at 1, 3, 7, and 14 days, respectively. The sixth group underwent a CFA injection in the TMJ and a simultaneous saline injection in the trigeminal ganglion. The seventh group received CFA in the TMJ and CGRP in the ganglion. The eighth group was administered CFA in the TMJ and CGRP 8-37 in the ganglion. For the administration of drugs via ganglion injection, after establishing an arthritis model in SD rats through CFA injection into the temporomandibular joint, drugs were administered into the trigeminal ganglion of the rats. Anesthesia was administered using 1.5% isoflurane in oxygen, sourced from Abbott Laboratories, North Chicago, IL, USA. Following the established protocol (Kameoka et al. 2010), a 27-gauge 0.5-inch needle was precisely inserted at the anterosuperior portion of the zygomatic arch root of the rat. We then administered a bilateral slow injection of 50 µL of either saline or CFA (product number F5881 from Sigma-Aldrich, USA) into the upper compartment of the TMJ.

### Intra-Ganglionic Drug Administration

**Fig. 1:**
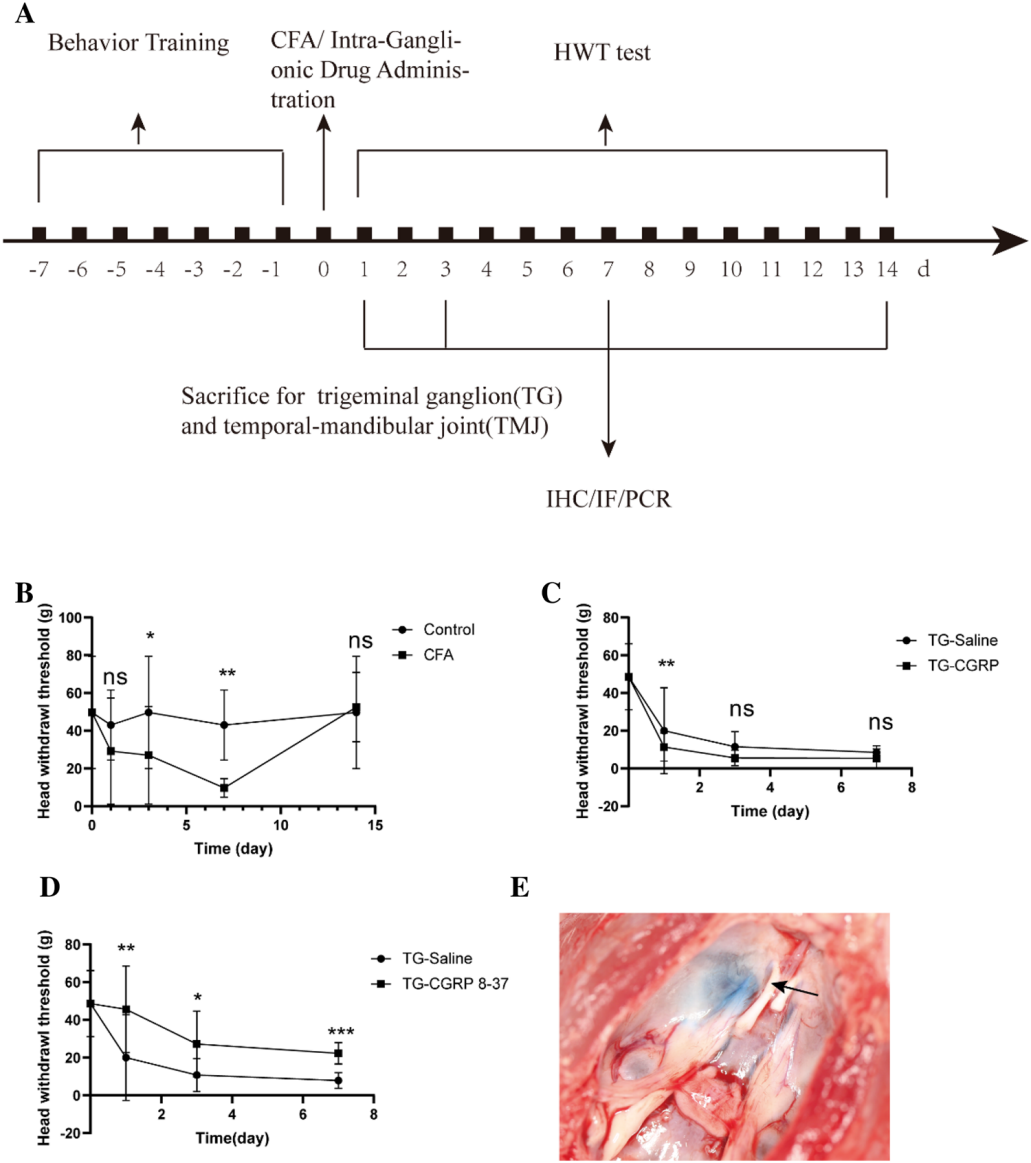
Temporomandibular Arthritis Pain Response in Rats. **A** Overview of the experimental setup. **B** Rat pain response post-CFA induction. **C** Behavioral outcomes following CGRP injection in the trigeminal ganglion. **D** Effects of CGRP 8-37 antagonist on pain behavior. **E** Illustration of drug administration targeting the trigeminal area. Data are presented as mean ± SD. The *t*-test was used for analysis; *n* = 6. Number of technical replicates (NTR) = 3. Significance: *p < 0.05, **p < 0.01, ***p < 0.001

In the CFA-injected group, rats were categorized into three subgroups for trigeminal ganglion injection: CGRP, CGRP 8-37, and normal saline, with 6 rats in each subgroup. We prepared the solutions of rat CGRP (10^−5^ M, MCE) and CGRP 8-37 (a CGRP antagonist, 10^−5^ M, MCE), both at a concentration of 10^–5^ M, in normal saline(Afroz et al. 2019). These were aliquoted and stored at − 20 °C in accordance with the manufacturer's guidelines. For TG intra-ganglionic (IG) drug delivery, we followed the previously published protocols (Neubert et al. 2005). A 26-gauge Hamilton syringe with a 10 µL capacity was used for IG administration. The entry point for the needle was the infra-orbital foramen, positioned 1 mm medial to the zygomatic process of the maxilla. We inserted the needle approximately 22 mm, angling it toward the midline by roughly 10 degrees and 15 degrees downward from the plane of the parietal bone (Villa et al. 2010). This procedure allowed the needle to pass through the infraorbital canal and reach the ipsilateral trigeminal ganglion located in Meckel’s cave. To verify the accuracy of our injections, methylene violet was injected to visualize the trigeminal ganglion (Fig. 1E).

### Behavioral Assessment

To assess mechanical allodynia in inflamed animals, we implemented a previously established protocol (Ren 1999), incorporating minor modifications for our specific needs. The study began by acclimatizing the rats to the test environment for one week. This was followed by a seven-day behavioral training period prior to establishing the temporomandibular arthritis model. During this phase, SD rats were placed unrestrained in a glass box situated in a quiet room, allowing them to adapt for 30 min. The next step involved measuring the pain threshold in the preauricular temporomandibular joint area of the SD rats using Von Frey filaments (Biological Instruments, North Coast Medical, America). Notably, while the rats’ heads were not restrained during the measurement, their overall movements were still limited. This procedure was focused on testing the orofacial skin regions adjacent to the center of the temporomandibular joint on both sides. Von Frey filaments were specifically employed to assess the TMJ mechanical sensitivity, with measurements conducted at 1, 3, 7, and 14 days post-injection. For mechanical sensitivity testing, Von Frey filaments were applied in ascending order, starting with a 2 g filament for control animals and a 0.16 g filament for inflamed animals. Each filament was applied five times at intervals of a few seconds, and the response threshold was determined by the minimal force that induced three or more head withdrawal reactions during the five applications. We maintained a consistent 2-min interval between subsequent filament applications. All testing procedures were conducted in a tranquil setting to preclude the influence of diurnal variations.

### Cell Culture and α-CGRP Treatment

The THP-1 human monocytic leukemia cell line, authenticated by short tandem repeat spectrometry, was acquired from Procell Life (Wuhan, China). It was cultured in RPMI 1640 medium (Procell Life, Wuhan) supplemented with 10% heat-inactivated fetal calf serum (FCS), and the cells were maintained at 37 °C in a humidified atmosphere containing 5% CO2. After cultivating, the THP-1 cells underwent centrifugation at 800 RPM/min for 5 min. Post centrifugation, the supernatant was removed, and the cell density was adjusted to 1 × 10^6^/mL using fresh culture medium. Subsequently, Phorbol ester (PMA) was introduced to the cell suspension until it reached a concentration of 100 ng/ml. Following gentle mixing, the cell suspension was allocated into 6-well plates, with each well containing 2 ml. These plates were then incubated under controlled light exposure. After a 48-h treatment with PMA, a notable change was observed in the cells: they transitioned from being in suspension to displaying protruding pseudopods as adherent cells. This transformation was confirmed under a microscope. Eventually, the THP-1 cells differentiated into M0 macrophages, which were then cleansed twice with sterile PBS. To understand the impact of CGRP on the inflammatory response of these macrophages, cells were pre-treated with CGRP (10^−6^ M, MCE) for 30 min. Thereafter, they were either cultured in a medium containing 50 ng/mL LPS (MCE) + 20 ng/mL IFN-γ(MCE) for 24 h or in another medium with 20 ng/mL IL-4(MCE) + 20 ng/mL IL-13(MCE), specifically for a 72-h period targeting M0 macrophages.

### Tissue Preparation

Rats were euthanized using an overdose of isoflurane for a humane endpoint. We meticulously extracted the temporomandibular joint tissues, including the mandibular condyle disc, retro-discal area, fossa, and the trigeminal ganglion, from each rat across all groups. These tissues were then fixed in 4% paraformaldehyde for a 24-h period. For histopathological evaluation, the TMJ specimens were subjected to demineralization in a 19% solution of EDTA, with the solution being refreshed every three days. Meanwhile, TG tissues were prepared for dehydration by immersion in 30% sucrose solution overnight.

### Quantitative Real-Time Polymerase Chain Reaction (q RT-PCR)

Total RNA was isolated from cells utilizing the RNAiso Plus reagent (TaKaRa), which was then reverse-transcribed into cDNA with TaKaRa’s reverse transcription kit. The quantitative real-time PCR (qRT-PCR) was performed using SYBR Green (TaKaRa) as the DNA-binding dye, on the 7500 Fast Real-Time PCR System (Applied Biosystems). For normalization purposes, the expression of β-actin mRNA was used as a reference against the expression of the target mRNA. For each cell sample, three replicate wells were set up to eliminate technical errors in the experiment. The specific sequences for the PCR primers utilized in this investigation were shown below: CD86 F_5ʹ-GTTTCATTCCCTGATGTTACGAG-3ʹ R_5ʹ-GAGAAAGGTGAAGATAAAAGCCG-3ʹ, CD206 F_5ʹ-GCTGAAAGGT. GACCCTACTATGT-3ʹ R_5ʹ-GCTCAGGTTTTGGTGTTTGTC-3ʹ.

### Histology

Demineralized TMJs from two groups (CFA-treated, *n* = 6 joints; Saline-treated, *n* = 6 joints) were embedded in paraffin and cut into 5 µm thick mid-sagittal sections. These sections were then dewaxed, rehydrated, and triple-washed with PBS to prepare for antigen retrieval. For this process, the slides were immersed in sodium citrate (pH 6.0) and heated to 95 ℃ for 20 min. Following antigen retrieval, the sections were washed in PBS for 10 min and blocked with 10% bovine serum albumin for one hour at room temperature. Subsequently, these tissues were exposed to primary antibodies: CD86 (Huabio, ER1906, 1:400) and CD206 (CST, 24595, 1:500) and incubated overnight at 4 ℃. Upon completion, after three PBS washes, the sections were introduced to a horseradish enzyme-labeled streptavidin solution (Abcam; dilution, 1:500) for an hour at room temperature. The final step involved incubation in diaminobenzidine (DAB, Sigma-Aldrich) for coloration and a counterstain using hematoxylin. In this experiment, the immunohistochemistry assay was performed in triplicate on joint tissue from each identical biological sample, with the results being averaged across these technical replicates.

### Immunofluorescence Staining (IF)

TG tissues were prepared by embedding them in an optimal cutting temperature compound (OCT) (Sakura, USA) and then sectioning them serially at a thickness of 10 μm. First, the specimens underwent a 10-min wash using phosphate-buffered saline (PBS). This was followed by antigen retrieval, which entailed heating the specimens in citrate using a microwave oven for 25 min. To prevent nonspecific binding, we treated the tissues with 5% bovine serum albumin (BSA) for 1 h at 37 ℃. Subsequently, the tissues were incubated with primary antibodies: CGRP (CST, ab81887, 1:1000), CD206 (CST, 24595, 1:500), and CD86 (ABclonal, A1199, 1:100) overnight at 4 ℃. As a next step, the tissues were treated with goat anti-rabbit Alexa Fluor 488 (CST, 4412s, 1:1000) secondary antibodies for 1 h at 37 ℃. For cell nuclei visualization, DAPI (Beyotime, C1006, 1:100) was added, with a 5-min incubation period. Images were captured using a fluorescence microscope (Carl Zeiss, Baden-Wurttemberg, Germany). Within the TG, enumerations of both CGRP-positive neurons and total neurons, which were labeled with DAPI, were conducted across five fields of each section. Two independent evaluators performed these counts, using a 20 × objective lens for CGRP-labeled immunofluorescence, and a 40 × objective lens for CD86 and CD206 labeled immunofluorescence. The data was assessed based on the count of positive cells and the intensity of immunofluorescence. In the technical replicates for both immunofluorescence and immunohistochemistry experiments, this study employed a consistent procedure, where the results for each biological sample were derived from the average of three identical assays. Image J software was employed for all immunofluorescence data analyses.

### Statistical Analyses

Statistical analyses were conducted utilizing GraphPad Prism version 9.5 and SPSS version 25. All results are expressed as mean ± standard deviation (SD). We employed one-way analysis of variance (ANOVA) followed by Dunnett’s *t*-test to compare means, assuming normality and homogeneity of variances across samples. For all identical experiments, the number of technical replicates (NTR = 3) is three to exclude technical errors. Levels of statistical significance were denoted as follows: *p < 0.05, **p < 0.01, ***p < 0.001, and ****p < 0.0001.

## Results

### Time-Course of Inflammation and Behavioral Assessment

We established the pain model of temporomandibular arthritis in rats by CFA injection and conducted behavioral monitoring. Initially, within 7 days post-injection, we documented a time-dependent increase in pain via HWT measurements (Fig. 1B). A significant reduction in HWT was observed immediately following the injection, with the lowest HWT recorded on the 7th day (Fig. 1C). In the later phase, beyond 7 days, HWT began to recover but was still lower than the saline-injected control rats. The HWT was notably reduced on days 1, 3, 7, and 14 post-injection when compared to controls (*p* = 0.2747, *p* = 0.0102, *p* = 0.0017, and *p* = 0.3946, respectively). Treatment with CGRP 8-37 in the ganglion resulted in a significant increase in HWT relative to saline injections (Fig. 1D), particularly on days 1, 3, and 7 (*p* = 0.0017, *p* = 0.049, *p* = 0.0005, respectively). On the other hand, CGRP injections without the antagonist led to decreased HWT at these same time points compared to the saline group (*p* = 0.0024, *p* = 0.3595, *p *= 0.0436, respectively). Overall, the CFA injection induced a time-dependent pattern of pain, peaking on day 7. The application of CGRP 8-37 effectively mitigated this pain.

### Cell Size Distribution and CGRP-Immunoreactivity of Trigeminal Neurons

**Fig. 2:**
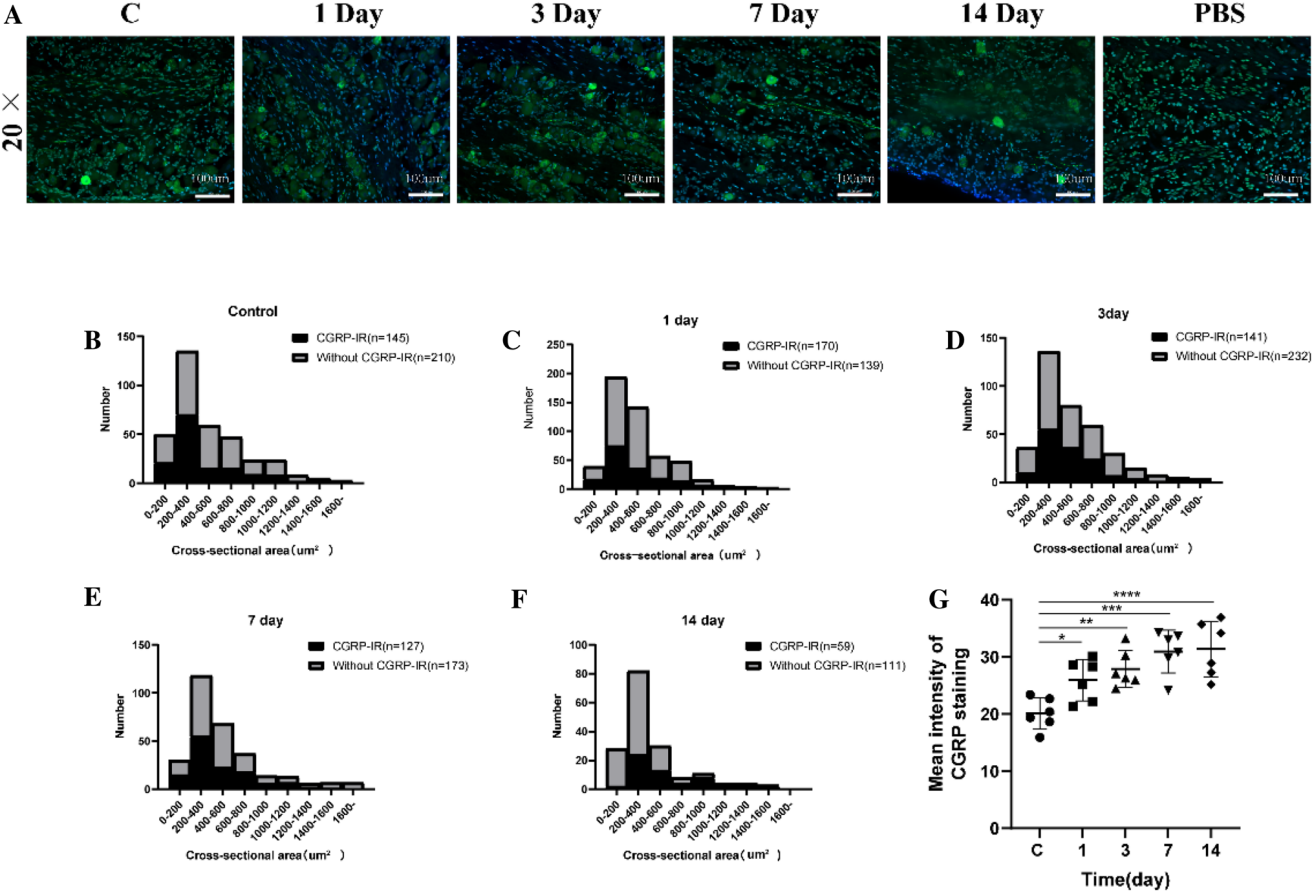
CGRP Expression Patterns in Trigeminal Nerve Post-Inflammation **A** Fluorescence of CGRP in rat trigeminal ganglia. **B**–**F** Comparative histograms of CGRP-immunoreactive cell sizes. **G** Fluorescence intensity in CGRP-labeled trigeminal nerves. Data mean ± SD, analyzed by ANOVA and Dunnett's *t*-test; *n* = 6. NTR = 3. Significance: *p < 0.05, **p < 0.01, ***p < 0.001, ****p < 0.0001

Immunofluorescence indicates that neurons labeled with CGRP are mainly found at the junction of the trigeminal ganglion and the mandibular branch. Concurrently, this study delineates the temporal changes in the size (μm^2^) of these neurons in the trigeminal ganglion following CFA-induced temporomandibular joint arthritis in rats (Fig. 2). A marked increase in the mean fluorescence intensity of CGRP compared to the control group was observed on days 1, 3, 7, and 14 post-induction (*p *= 0.0372, *p* = 0.0044, *p* = 0.001, and *p* < 0.001, respectively), with the most pronounced changes on days 7 and 14 (Fig. 2G). Notably, CGRP-labeled trigeminal neurons in cases of temporomandibular arthritis were predominantly within the 200 to 400 μm^2^ range (Fig. 2B–F). These findings underscore the crucial involvement of CGRP-labeled neurons in the development of temporomandibular arthritis pain and their impact on both the peripheral and central nervous systems.

### The CD206/CD86 Expression in the Trigeminal Ganglion of Rats After CFA-Induced TMJ OA

**Fig. 3:**
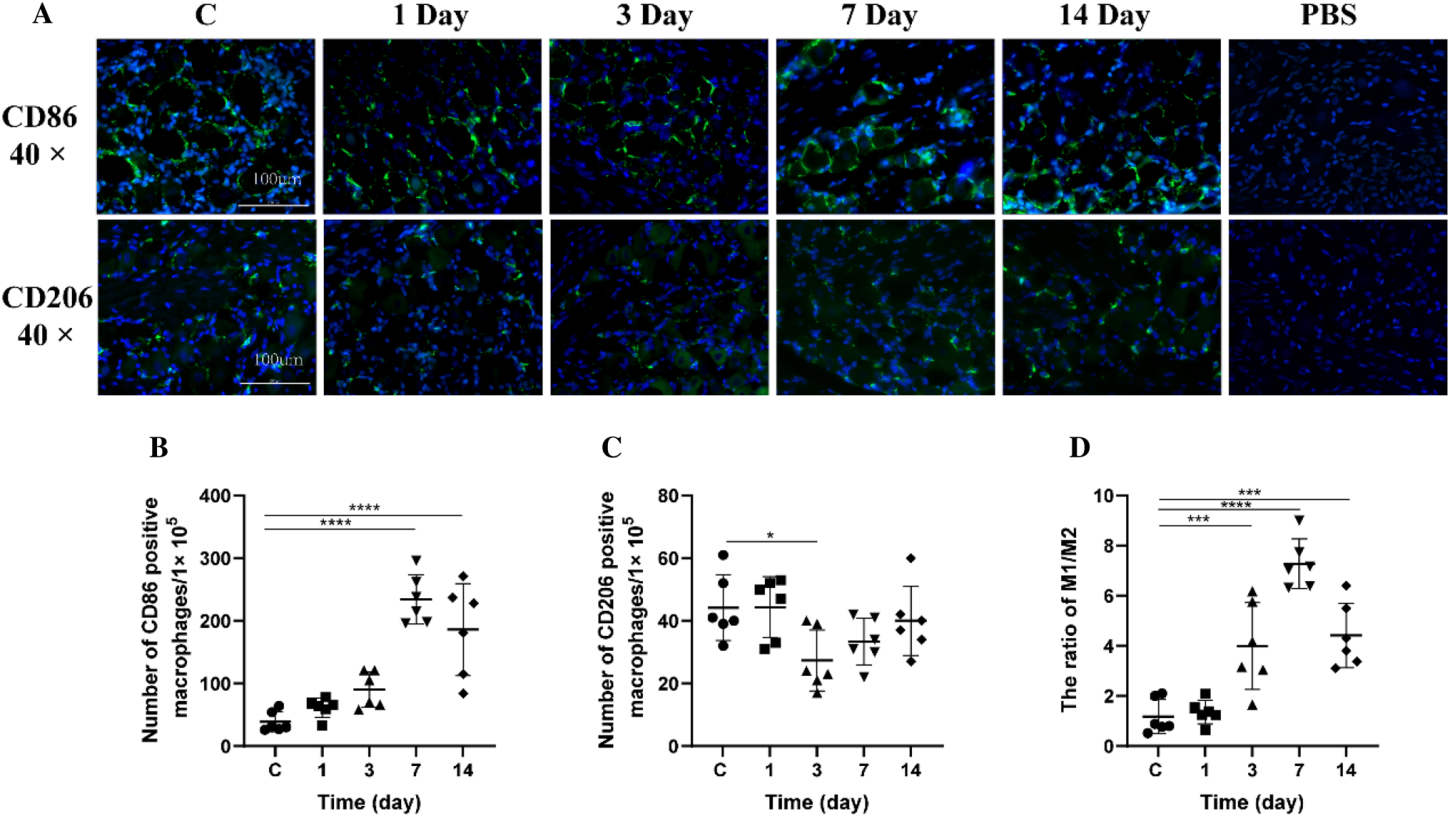
Dynamics of CD86/CD206 in Trigeminal Ganglion Post-CFA Injection **A** Immunofluorescence of CD86/CD206 in trigeminal ganglia. **B** Count of CD86 + macrophages. **C** Count of CD206 + macrophages. **D** M1/M2 macrophage ratio. TG denotes trigeminal ganglion. Data presented as mean ± SD, analyzed via ANOVA and Dunnett’s *t* test; *n* = 6. NTR = 3. Significance: *p < 0.05, **p < 0.01, ***p < 0.001, ****p < 0.0001

To assess the changes in M1 and M2 macrophage populations in the trigeminal ganglion after CFA-induced temporomandibular joint arthritis, we examined the expression of CD206 and CD86 in the TG using immunofluorescence in frozen rat sections (Fig. 3A). We identified both CD206-positive M2 and CD86-positive M1 macrophages in the TGs of both naive and CFA-treated rats. Quantitative analysis showed an increase in CD86-positive M1 macrophages post-CFA treatment, peaking at day 7 (Fig. 3B). These macrophages increased progressively on days 1, 3, and 7 compared to the control group, with the peak at day 7 and a subsequent decrease by day 14 (*p* = 0.7376, *p* = 0.1193, *p* < 0.0001, and *p* < 0.0001, respectively). In contrast, the number of CD206-positive M2 macrophages declined within 24 h after CFA treatment, reaching the lowest level on day 3, and began to recover, returning to baseline levels by day 7 (Fig. 3C). This trend was significant on day 3 (*p* = 0.0218) but not on other days when compared to the control group. The M1/M2 macrophage ratio in the TG rose until the end of the first week and then decreased by the end of the second week (Fig. 3D), with the ratio changes being statistically significant on day 3 and day 7 (*p* = 0.0008, *p* < 0.001, respectively). Notably, CD206-positive M2 macrophages were observed surrounding TG neurons, displaying distinct morphology and size compared to the neurons. These findings highlight a possible link between CGRP-expressing neurons and macrophage polarization, which may play a role in the modulation of neuroinflammation.

### Intra-Ganglion Injection of CGRP-Induced Macrophage Polarization to M2

**Fig. 4:**
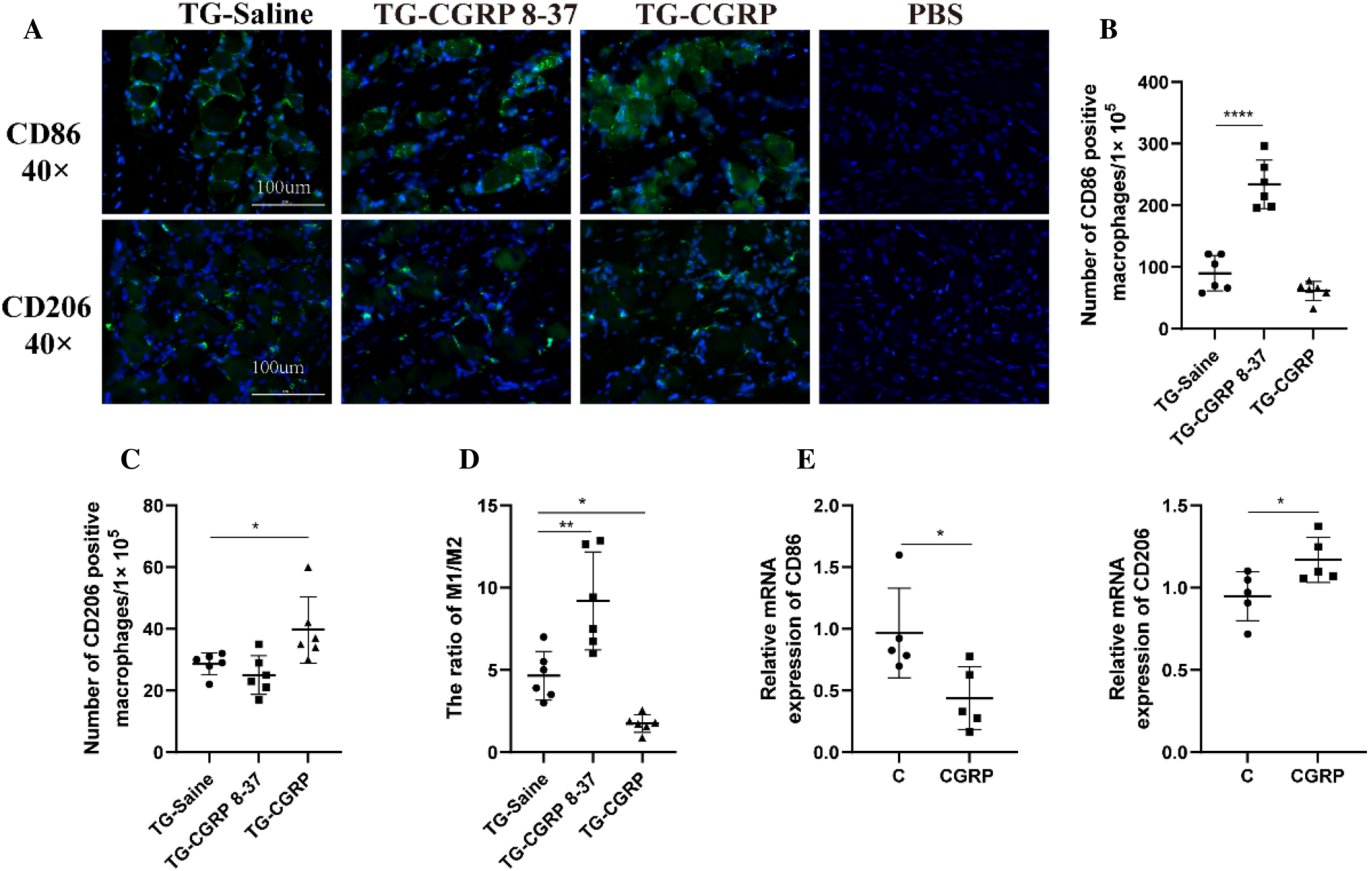
Impact of CGRP and Antagonist on Macrophage Markers **A** Immunofluorescence images labeled for CD86/CD206. **B** CD86 expression in macrophages after CGRP and antagonist treatment. **C** CD206 expression changes following treatment. **D** Alteration in the M1/M2 macrophage ratio due to treatments. **E** In vitro effects of CGRP on CD86/CD206 expression. Data are mean ± SD, analyzed with ANOVA and Dunnett’s *t* test; *n* = 6. NTR = 3. Significance: *p < 0.05, **p < 0.01, ***p < 0.001, ****p < 0.0001

To explore the influence of CGRP on macrophage polarization in the TG, we administered CGRP and its antagonist, CGRP 8-37, directly into the TG. The application of CGRP promoted a shift in macrophages towards the M2 phenotype compared to the control. Conversely, the administration of CGRP 8-37 impeded this M2 polarization (Fig. 4B, C). Relative to the control, the M1/M2 ratio notably increased following the injection of CGRP 8-37, with significant statistical differences (Fig. 4D). After the TG was injected with CGRP 8-37 and CGRP, CD86-positive macrophages were significantly increased and decreased, respectively (*p *< 0.001 and *p* = 0.1939, respectively), while CD206-positive macrophages exhibited an inverse pattern (*p* = 0.6133 and *p *= 0.0403, respectively). The ratio of CD86-labeled M1 to CD206-labeled M2 macrophages correspondingly increased and decreased (*p* = 0.002 and *p *= 0.0037, respectively). Moreover, in vitro studies corroborated that CGRP induces a transition towards the M2 phenotype and deters the transition towards the M1 phenotype under inflammatory conditions (Fig. 4E). Specifically, CGRP treatment inhibited the differentiation of M0 macrophages into M1 macrophages and facilitated their differentiation into M2 macrophages (*p* = 0.0280 and *p* = 0.0226, respectively), confirming the modulatory role of CGRP in macrophage polarization.

### CD86/CD206/CGRP Expression in TMJ Tissue of Rats with CFA-Induced TMJ-OA

**Fig. 5:**
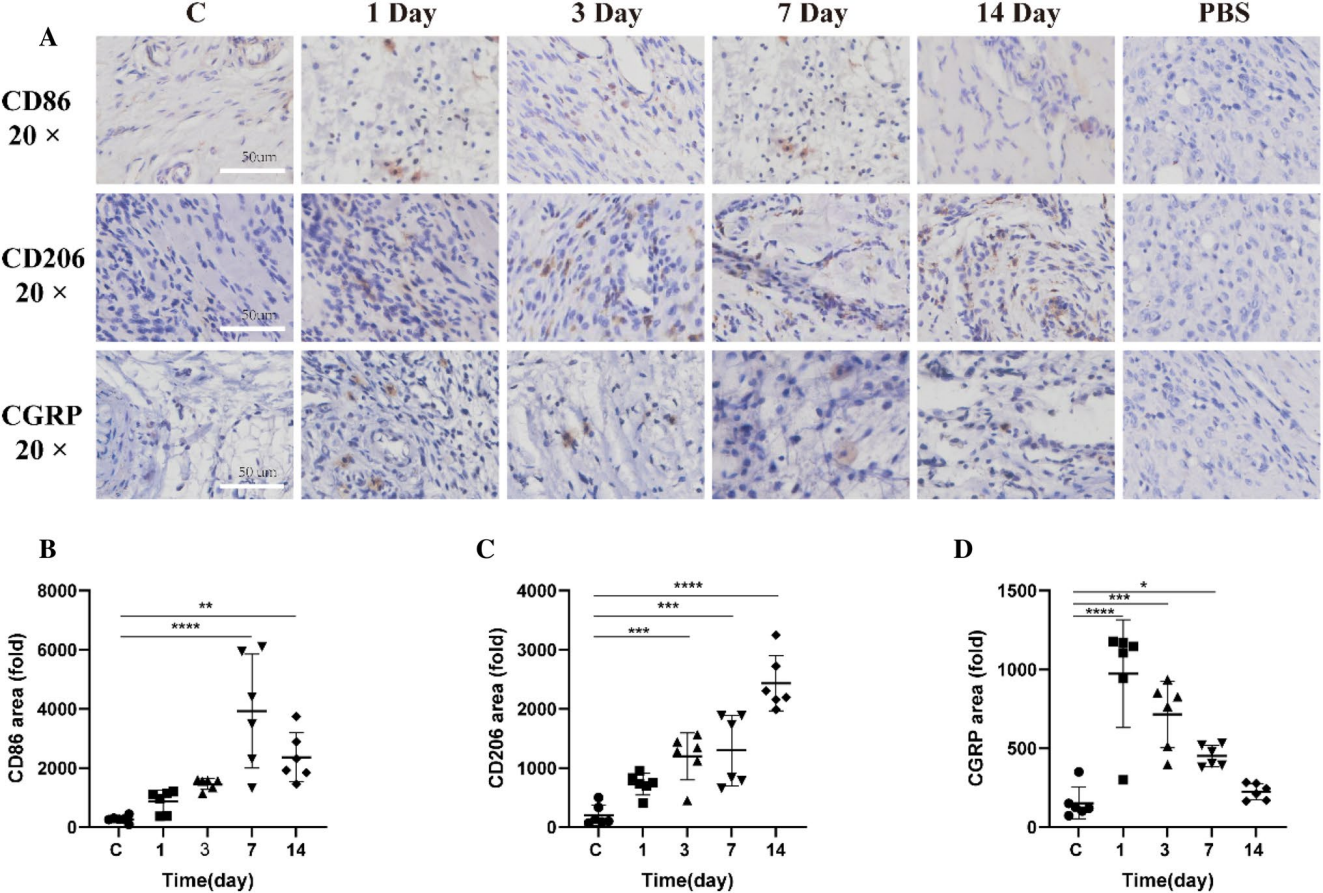
Temporal Dynamics and Distribution of CD86, CD206, and CGRP in the Temporomandibular Joint's Soft Tissue Post-CFA-Induced Inflammation. **A** Depicts immunohistochemical localization of CD86, CD206, and CGRP. **B** Highlights CD86 expression. **C** Details CD206 expression. **D** Illustrates CGRP expression. Data are presented as mean ± SD, analyzed via one-way ANOVA with Dunnett’s *t* post hoc test. Sample size (*n*) = 6. NTR = 3. Notations of significance: *p < 0.05, **p < 0.01, ***p < 0.001, ****p < 0.0001

To elucidate the M1/M2 macrophage dynamics in temporomandibular joint tissues affected by CFA-induced inflammation, immunohistochemistry was used to assess CD86, CD206, and CGRP expressions in rat TMJ (Fig. 5A). Both control and CFA-treated rats displayed CD86-positive M1 and CD206-positive M2 macrophages in the TMJ tissues. Notably, CD86-positive M1 macrophages significantly increased one day after CFA treatment, peaking at day 7 (Fig. 5B). These macrophages in the TMJ's surrounding soft tissues surged initially and then diminished from day 14 onwards (*p* = 0.6672, *p* = 0.1276, *p* < 0.0001, *p* = 0.0033, respectively). Likewise, CD206-positive M2 macrophages increased, reaching a maximum at two weeks post-inflammation (Fig. 5C). Their numbers rose progressively on days 1, 3, 7, and 14 (*p* = 0.0999, *p *= 0.0008, *p* = 0.0003, *p *< 0.0001, respectively). CGRP expression, primarily around blood vessels, significantly spiked one day post-CFA and then declined (Fig. 5D), with the highest expression on day 1, decreasing on days 3, 7, and 14 (*p* < 0.0001, *p* = 0.0001, *p* = 0.0381, *p* = 0.9095, respectively). These observations demonstrate the critical role of the M1–M2 macrophage shift in the inflammation and progression of TMJ arthritis.

### The Effect of CGRP on Pathological Process and CD206/CD86 Expression in TMJ Tissue of Rats with CFA-Induced TMJ-OA

**Fig. 6:**
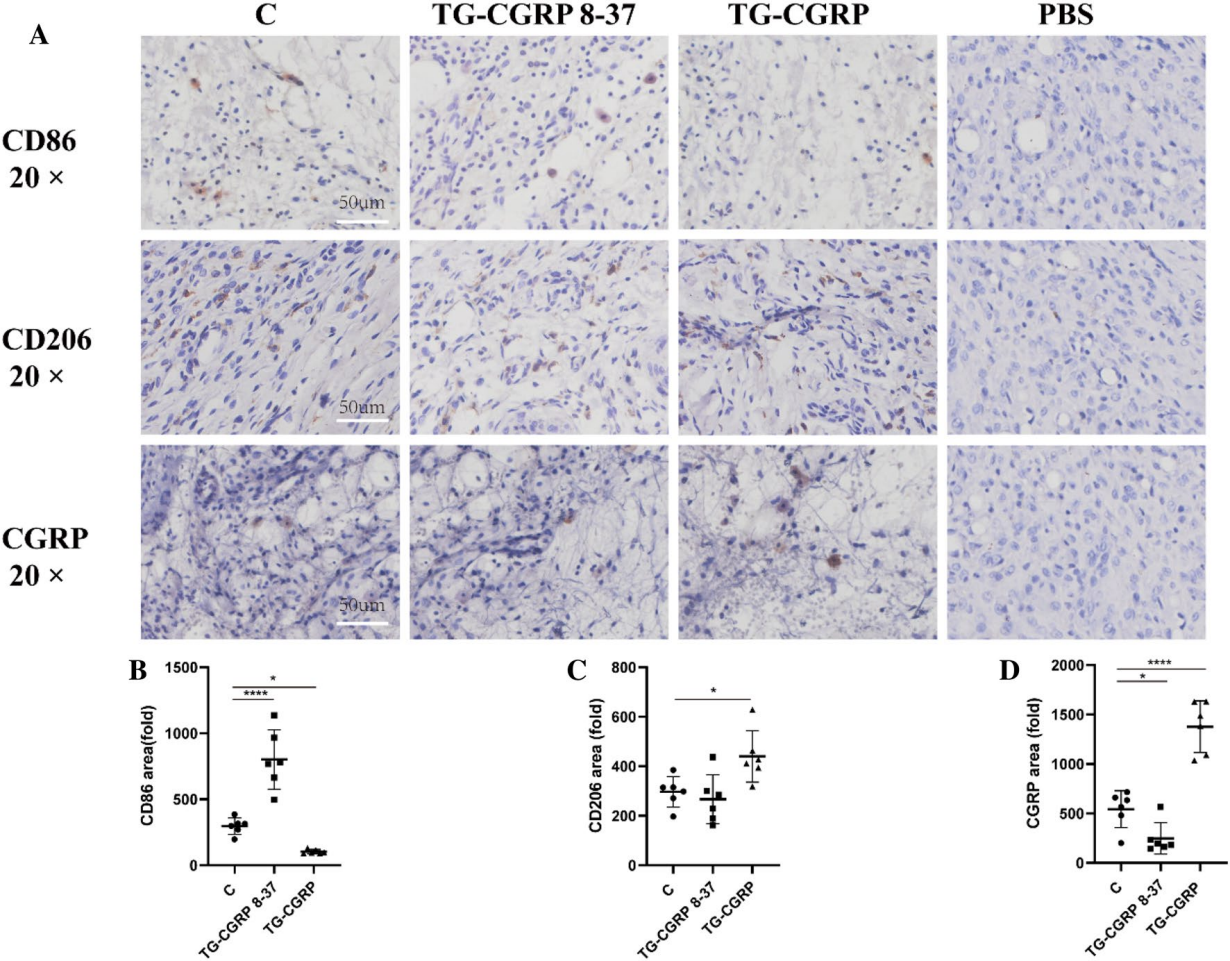
Influence of IG CGRP and CGRP 8–37 on CD86/CD206/CGRP in TMJ soft tissue: **A** Immunohistochemistry for CD86/CD206/CGRP; **B** Changes in CD86 post-injection; **C** CD206 expression shifts; **D** CGRP protein alterations post-treatment. Data are represented as means ± SD and were analyzed by one-way analysis of variance (ANOVA) followed by Dunnett’s *t* test. Sample size (*n*) = 6; NTR = 3. Significance levels indicated as: *p < 0.05, **p < 0.01, ***p < 0.001, ****p < 0.0001

To assess the impact of CGRP and CGRP 8-37 on macrophage polarization in the temporomandibular joint vicinity, we administered these compounds into the trigeminal ganglion of rats with CFA-induced TMJ arthritis. Using immunohistochemistry, we detected CD86, CD206, and CGRP expressions, as shown in Fig. 6A. CGRP 8-37 injections significantly increased CD86 levels in the TMJ adjacent tissues, with no notable change in CD206 levels and a reduction in CGRP compared to the control (Fig. 6B–D). In contrast, exogenous CGRP injections decreased CD86 levels while increasing CD206 and CGRP expressions significantly. Specifically, the number of CD86-positive M1 macrophages rose with CGRP 8-37 and fell with CGRP treatment (*p* < 0.0001, *p* = 0.045, respectively), while CD206-positive M2 macrophages showed the opposite trend (*p* = 0.7932, *p* = 0.0272, respectively). CGRP levels followed a similar pattern, decreasing with CGRP 8-37 and increasing with CGRP injection (*p* = 0.0467, *p* < 0.0001, respectively). These results underscore the regulatory effects of CGRP on macrophage polarization, which may contribute to the pathophysiology of TMJ arthritis.

## Discussion

This study pioneered an exploration into the temporal dynamics and spatial localization of CGRP expression within the trigeminal ganglion and the regulation of the M1/M2 macrophage balance throughout the progression of TMJ in rats. We observed that as the model induction time increased, CGRP expression in both the trigeminal ganglion and the temporomandibular joint progressively increased, correlating with heightened pain behavior. Triggered by peripheral inflammation and nerve injury, there is an upregulated release of CGRP from neurons in the trigeminal node. When exogenous CGRP is administered directly into the trigeminal ganglion in vivo, there's notable promotion in the macrophages’ transformation into M2 within the trigeminal ganglion, accompanied by an augmented CGRP expression in the temporomandibular joint tissues. Contrastingly, an injection of the CGRP receptor-specific inhibitor, CGRP 8-37, into the trigeminal ganglion relieved TMJ pain and concurrently lowered the CGRP expression levels in peripheral TMJ tissues.

CGRP-positive neurons are notably prevalent within the TMJ complex, including areas like the joint capsule, synovial membrane, temporomandibular joint posterior disc tissue, masticatory muscles, tendons, and fascia (Azuma and Sato 2017; Lindquist et al. 2021; Suttle et al. 2023). Echoing our findings, CGRP was predominantly expressed around the blood vessels in the temporomandibular joint tissues of rats. Known for its extensive presence in both central and peripheral nervous systems and in pain pathways of unmyelinated A δ and C sensory nerve fibers(Chen and Wu 2020; Iyengar et al. 2019), CGRP is chiefly discharged from peripheral nerve fibers, such as those found in the trigeminal ganglion. Originating from the cell bodies of sensory neurons, it is then conveyed to their peripheral endpoints (Ulrich-Lai et al. 2001).

CGRP is prominently expressed in the trigeminal nervous system, with its levels notably rising in the trigeminal ganglion during the chronic pain phase of OA (Appelgren et al. 1995; Jiang et al. 2022; Miller et al. 2017). This molecule, while not directly instigating the disease's acute inflammatory process, is pivotal in modulating inflammation. Supporting its significance, research indicates that the specific CGRP receptor antagonist MK-8825 can substantially mitigate facial pain and curtail the widespread release of pro-inflammatory cytokines (Romero-Reyes et al. 2015). These findings align with our experimental study results. When CFA is administered into the temporomandibular joint, there’s not only an upregulation of CGRP expression in the sensory system of trigeminal nerve injury but also an augmentation in the peripheral distribution of CGRP within the temporomandibular joint—a phenomenon closely linked to pain behavior. In the TG context, utilizing the CGRP antagonist CGRP 8-37 has shown pain-relieving effects. Yet, the introduction of exogenous CGRP in the TG leads to diminished peripheral pain thresholds, accompanied by enhanced CGRP expression in the vicinity of the temporomandibular joint tissues.

Macrophages located within sensory ganglia are pivotal in regulating inflammation, facilitating nerve tissue repair, and modulating neuropathic pain post-peripheral nerve injury. Existing research has highlighted the swift proliferation of ganglionic tissue-resident macrophages after such injuries. These macrophages infiltrate the spaces between satellite glial cells and neurons, directly engaging with the latter. Characteristically behaving as the M2 type and with minimal neuronal cell death, it is inferred that this neuron-macrophage interaction is instrumental for tissue repair (Iwai et al. 2021). In this study, our analysis indicated a rise in CD86-labeled M1 macrophages within the trigeminal ganglion during acute inflammation following the intra-TMJ injection of CFA. Concurrently, there was an initial decline followed by an upsurge in CD206-labeled M2 macrophages. This dynamic played a pivotal role in preserving the inflammatory homeostasis within the trigeminal ganglion. Intriguingly, the elevation in CGRP expression within the trigeminal ganglion correlated with an increase in M2-labeled macrophages. When exogenous CGRP was injected into the TG, the count of CD206-labeled M2 macrophages rose, while CD86-labeled M1 macrophages decreased. Furthermore, the introduction of exogenous CGRP into the trigeminal ganglion enhanced CGRP expression in the surrounding TMJ tissues. This was accompanied by a rise in CD206-labeled macrophage expression in adjacent TMJ tissues and a reduction in CD86-labeled macrophages. However, the administration of CGRP 8-37 diminished both CGRP expression and TMJ pain, with a concurrent relative reduction in CD206-labeled macrophages within TMJ tissues.

In this study involving CFA-induced TMJ, both M1 and M2 macrophages in the trigeminal ganglion predominantly encircle CGRP-labeled neurons. This arrangement suggests a modulatory role of CGRP in macrophage activation and polarization within the ganglion. Macrophages have the capability to navigate via diverse signaling routes, adapting to microenvironmental changes. Numerous research studies have characterized the subset of macrophages responsive to tissue injury as “activated macrophages”. While their appropriate activation is crucial for upholding physiological harmony in the body, any aberrant activation could induce tissue impairment and immune dysfunctions, leading to various ailments. It is documented that macrophages are instrumental in the onset, progression, and development of nerve injuries and peripheral sensitization, a trait attributed to the profound functional plasticity of these cells (T’Jonck et al. 2018; Zhang et al. 2016).

CGRP has been identified to have anti-inflammatory and immunoregulatory roles in inflammatory responses. Serving as key modulators for tissue stability and repair, macrophages can switch their phenotype from pro-inflammatory to either anti-inflammatory or pro-healing (Körner et al. 2019). Consistent with this, research has demonstrated that CGRP can specifically influence this phenotypic transition, promoting IL-4-induced M2 macrophages while inhibiting LPS-induced M1 macrophages (Duan et al. 2017; Yuan et al. 2020). In vitro, this experiment corroborates these findings, showing that CGRP, a neuropeptide secreted by the trigeminal ganglia, effectively facilitates the transformation of M1 macrophages into M2 macrophages. Extending this work into an in vivo context, this study in rats with temporomandibular arthritis confirms that CGRP helps guide the polarization of macrophages toward an M2 phenotype in both the trigeminal ganglia and the surrounding tissues of the temporomandibular joint, thereby contributing to the alleviation of inflammation and restoration of tissue homeostasis.

**Fig. 7:**
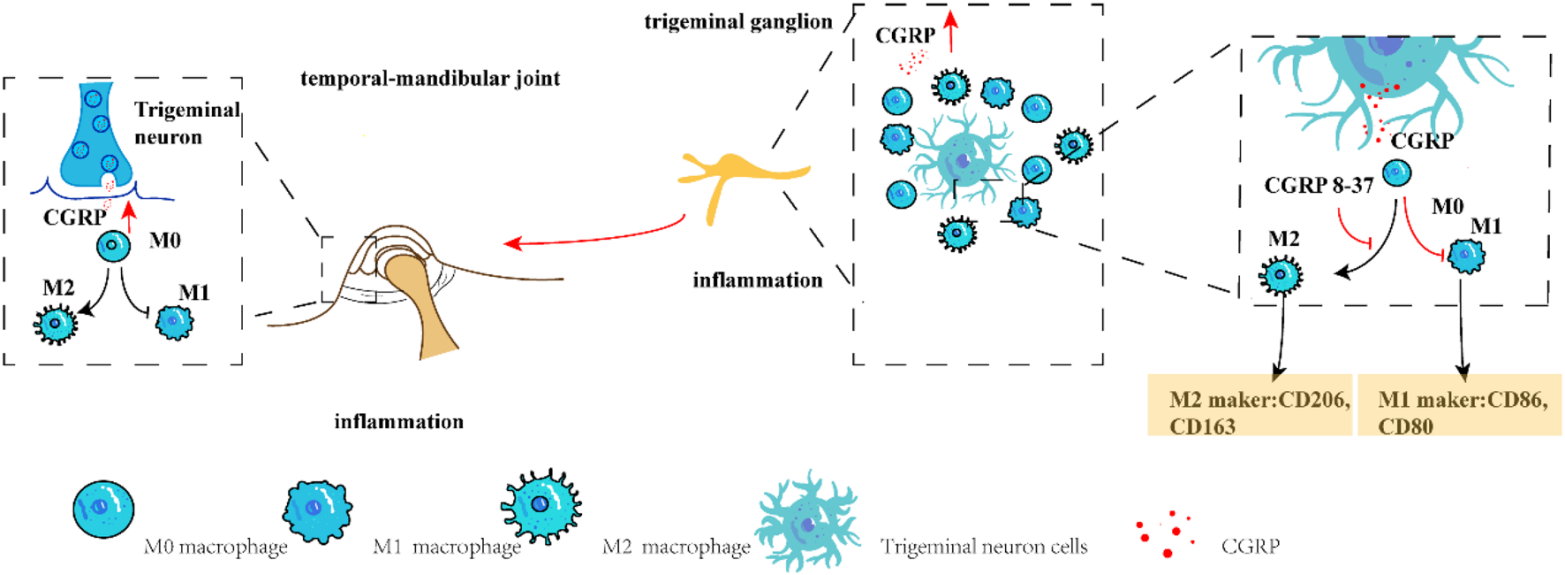
Schematic diagram showing CGRP expression and macrophage polarization induced after intra-articular injection of CFA

Although our study employs a model representative of the acute phase of TMD, it's notable that TMD is largely characterized as a chronic condition (Jiang et al. 2022; Renton 2020; Romero-Reyes and Uyanik 2014). While the impacts of CFA are traditionally examined over extended periods to monitor chronic inflammatory reactions, our findings demonstrate that within just 24 h, macrophages in the primary sensory nerve center, the trigeminal ganglion, initiate polarization. This evidence suggests that, from the onset, CGRP is pivotal in mediating nociceptive responses by influencing macrophage polarization. The clinical relevance of these insights cannot be understated: even though TMD primarily presents as a chronic issue, acute episodes often surface following extensive dental procedures and risk transitioning to chronic stages if unmanaged. To our understanding, this constitutes the inaugural investigation into the consequences of exogenous CGRP injections within the trigeminal ganglion concerning pain perception related to temporomandibular arthritis, and the influence of CGRP on macrophage polarization in the same region. In our comprehensive analysis of the research results, we found that in cases of rat temporomandibular arthritis, an increased CGRP release in the trigeminal ganglion correlates with a heightened expression of CGRP in the tissues surrounding the temporomandibular joint. Furthermore, macrophages in the TG undergo significant phenotypic transformations, which are pivotal in the initiation and progression of temporomandibular arthritis (Fig. 7).

## Conclusion

In rats with CFA-induced temporomandibular arthritis, we observed a significant recruitment and polarization of macrophages within the trigeminal system. Delving into the findings, the study primarily reveals two major conclusions:

- 1. Within the trigeminal ganglion, the presence of CGRP facilitates the conversion of macrophages to the M2 phenotype, effectively mitigating the inflammatory processes.
- 2. Macrophages are integral to the neuroinflammatory response. Significantly, their phenotypic changes within the TG are pivotal in initiating and further driving the progression of temporomandibular arthritis.

## Data Availability

The datasets utilized and analyzed during the current study are available from the corresponding author upon reasonable request.
